# Antihypertensive therapy to prevent cardiac death: A study of combined ACE inhibitors and β-blockers—a retrospective cohort study in Tsunan Town, Japan

**DOI:** 10.1371/journal.pone.0328142

**Published:** 2025-10-24

**Authors:** Shinichiro Ishikawa, Yusaku Hayashi, Toshiyuki Abe, Susumu Tanaka

**Affiliations:** 1 Department of Cardiovascular Medicine, Jikei University School of Medicine, Minato, Tokyo, Japan; 2 Department of Internal Medicine, Tsunan Town Hospital, Tsunan, Niigata, Japan; 3 Former Tokamachi Public Health Center, Tokamachi, Niigata, Japan; 4 Former Niigata Prefectural Welfare and Health Department, Health and Welfare Division, Niigata, Niigata, Japan; Universiti Tunku Abdul Rahman Fakulti Perubatan dan Sains Kesihatan M Kandiah, MALAYSIA

## Abstract

Antihypertensive treatment is widely known to reduce the risk of cardiovascular mortality; however, its protective effect, specifically against cardiac death, remains unclear. In this study, we examined whether a treatment strategy prioritizing the combined use of angiotensin-converting enzyme inhibitors and β-blockers reduces the risk of cardiac death in outpatient hypertensive patients. This retrospective observational cohort study was conducted at a single facility over a 30-year period, using data obtained between 1987 and 2016. Between 1992 and 2001, a combined treatment approach using angiotensin-converting enzyme inhibitors and β-blockers was preferentially used to suppress neurohumoral factors, with calcium channel blockers and diuretics used as supplementary medications. Standardized mortality ratios for all-cause mortality, cardiac death, and cerebrovascular death during each period were tracked and compared with nationwide data in Japan. Since 1992, the standardized mortality ratios for all-cause mortality and cardiac death in Tsunan Town have significantly decreased and fallen below the national averages. The present study focused on the role of neurohumoral factors, and we observationally evaluated the impact of combined therapy with angiotensin-converting enzyme inhibitors and β-blockers on the prognosis of patients with hypertension. While providing a perspective that has not been sufficiently examined to date, our findings should be regarded as the generation of an important hypothesis that warrants confirmation through future rigorous interventional studies.

## Introduction

The primary objective of hypertension treatment is to “reduce all-cause mortality,” as stated in the Japanese Society of Hypertension Guidelines [1]. The current treatment policy for hypertension is fundamentally to lower blood pressure [2], regardless of the type of medication used. Strict blood pressure control significantly reduces all-cause and cardiovascular mortality and is recommended in many guidelines [1–3].

However, it has been reported in several meta-analyses that the reduction in all-cause mortality associated with intensive blood pressure control, compared with that of standard blood pressure management, is mainly due to a decrease in cerebrovascular deaths, with no significant effect on cardiac mortality [4]. Furthermore, findings from another meta-analysis revealed that intensive blood pressure control does not reduce all-cause or cardiovascular mortality [5].

Reportedly, differences in all-cause and cardiovascular mortality may occur depending on the type of antihypertensive drugs, even with equivalent blood pressure reduction [6]. In particular, it is considered that angiotensin-converting enzyme (ACE) inhibitors suppress all-cause mortality and cardiac death [7]. This effect has attracted attention as a “beyond blood pressure-lowering effect,” suggesting that in addition to blood pressure reduction, mechanisms such as suppression of neurohumoral factors may be involved.

Reports from observational studies suggest that cardiac deaths (especially myocardial infarction and sudden death) frequently occur in the early morning hours, during which activation of neurohumoral factors is implicated [8–10]. ACE inhibitors suppress the renin–angiotensin system; hence, it is believed that they suppress cardiac death. Meanwhile, the effect of β-blockers remains controversial [11,12].

The choice of concomitant medications is also important. Diuretics and calcium channel blockers (CCBs) activate the sympathetic nervous and renin–angiotensin systems, potentially weakening the effects of ACE inhibitors and β-blockers [13–16]. Intervention studies in patients with heart failure have shown significant prognostic improvement with combined ACE inhibitor and β-blocker therapy [17,18]. While no studies have investigated the prognosis associated with this combination therapy in patients with hypertension, some editorials and commentaries recommend its use [19].

In this study, we examined the effect of combined ACE inhibitor and β-blocker therapy on the prevention of cardiac death in patients with hypertension. Based on the hypothesis that suppression of neurohumoral factors and blood pressure reduction are important for preventing cardiac death, we prioritized combination therapy with ACE inhibitors and β-blockers, adding CCBs or diuretics as needed. The effectiveness of this treatment approach was evaluated in a retrospective observational study.

## Materials and methods

### Study design and participants

The focus of this retrospective cohort study was on all residents of Tsunan Town, Niigata Prefecture, Japan (population: approximately 10,000). Tsunan is an agricultural mountainous area. The population decreased from 12,955 in 1990–10,029 in 2015, whereas the proportion of older adults aged ≥65 years increased from 23.0% to 39.0% [20]. Medical facilities in the town included Tsunan Town Hospital and two clinics, with initial treatment for acute cardiac disease mainly provided by the town hospital.

Patients with hypertension were defined as those continuously visiting the hospital with confirmed medical and pharmacy prescription records of treatment for hypertension. As outpatient prescriptions were not issued outside the hospital, prescription records from the hospital pharmacy were used to ascertain antihypertensive drug usage. Patients who were prescribed antihypertensive drugs for reasons other than hypertension were excluded after reviewing their outpatient medical records. The study period was from 1987 to 2016. Newly diagnosed outpatients with essential hypertension—defined as a morning home systolic blood pressure of at least 135 mmHg—were the target of the study from 1992. Wherever possible, treatment was initiated with a combination of an ACE inhibitor and a β-blocker. Cases of secondary hypertension, such as primary aldosteronism or renal artery stenosis, were excluded. Additionally, patients who had already been prescribed other antihypertensive drugs and were subsequently switched (when possible) to the combination of an ACE inhibitor and a β-blocker were included. Antihypertensive drugs that were contraindicated in any of the patients were also excluded.

### Clinical outcomes and endpoints

Mortality from all causes, heart disease, cerebrovascular disease, and malignant neoplasms over time was assessed using objective cause-specific mortality statistics regularly reported by the Ministry of Health, Labor and Welfare and local governments [21]. The primary endpoints were 5-year all-cause mortality and death from heart or cerebrovascular disease. Secondary endpoints included death from malignant neoplasms. Death from heart disease was defined using International Classification of Diseases (ICD)-10 codes I20–I52, which included those from angina, myocardial infarction, chronic ischemic heart disease, and heart failure, among others, according to Ministry of Health statistics. The standardized mortality ratio (SMR) was used as the evaluation index.

Adjustment for confounding was performed using the baseline demographic data on age and sex distribution for the national and Tsunan Town population, provided in S1 Table. The results showed no significant discrepancies. Bias from differences in age structure was eliminated using the SMR [22].

### Statistical analyses

SMRs and their 95% confidence intervals (CI) were calculated and compared with the national average (set as 1.0) [22]. The calculation was performed using the Niigata Prefecture Medical Representative Calculation Sheet (version 0.71), developed by Susumu Tanaka. Population counts used total residents.

### Antihypertensive drug usage ratios

Nationwide antihypertensive drug usage ratios were cited—with permission—from data published by the Intercontinental Medical Statistics Health Inc. [23], IQVIA [24], and the National Database Open Data [25]. The drug quantity used at the hospital was annually calculated using the total price of all antihypertensive drugs prescribed, from pharmacy prescription records, matching national publication years.

### Treatment protocol

The combination of ACE inhibitors and β-blockers was the initially prioritized antihypertensive drug; however, CCBs and diuretics were added if blood pressure targets were not met. This sequence was based on the results from the ACCOMPLISH trial, which showed that the ACE inhibitor and CCB combination reduced cardiovascular death more than the ACE inhibitor and diuretic did [26].

This regimen was called the “ABCD strategy” (A = ACE inhibitors, B = β-blockers, C = CCBs, and D = diuretics), implemented between 1992 and 2006. Thereafter, treatment was at the discretion of the attending physician. The 5-year period before implementation (1987–1991) served as a control (period 0), with observational periods divided into six 5-year segments: period 0 (1987–1991), period 1 (1992–1996), period 2 (1997–2001), period 3 (2002–2006), period 4 (2007–2011), and period 5 (2012–2016). Changes in mortality indicators per period were retrospectively analyzed and compared with those observed in the national data.

The details of the drugs used are as follows:

- ACE inhibitors shown to reduce fatal events included enalapril (maximum dose, 10 mg/day) [7], trandolapril (2 mg/day) [27], and perindopril (8 mg/day) [7].
- β-blockers with proven mortality reduction included metoprolol (120 mg/day) [28], carvedilol (20 mg/day) [29], and bisoprolol (5 mg/day) [30]. These β-blockers were not used in hospitalized patients with decompensated heart failure (defined following the 2025 Guidelines of the Japanese Circulation Society/Japan Heart Failure Society [31]). For compensated heart failure (stage B or C, New York Heart Association classifications I–II, brain natriuretic peptide level >100 pg/mL), low-dose titration protocols from heart failure trials were applied [28–30].
- Short-acting CCBs were avoided owing to increased cardiac death risk; instead, long-acting dihydropyridine derivatives, such as amlodipine, were chosen [32].
- Diuretics were chosen based on the patients’ serum potassium level: for K+ < 4.5 mEq/L, spironolactone (50 mg/day) was used [33]; if side effects occurred (gynecomastia), it was substituted with eplerenone [34]. For K+ ≥ 4.5, indapamide (2 mg/day) was used instead of thiazide diuretics, as indapamide was reported to have better cardiovascular outcomes [35].

Since the late 1990s, angiotensin II receptor blockers (ARBs) have been widely used in Japan. These agents, similar to ACE inhibitors, are renin-angiotensin system inhibitors; nevertheless, their effectiveness in reducing fatal events was not established. Therefore, their use was generally avoided unless for specific reasons [19,36].

The treatment goal was to achieve an early-morning home systolic blood pressure of <135 mmHg. Because combination therapy also benefits those with mild hypertension [37], a low-dose ACE inhibitor and β-blocker combination was initiated. Medications were generally administered at bedtime based on evidence that better antihypertensive effect and cardiovascular death reduction are observed at night than at other times [38], despite recent data suggesting timing has no effect on cardiovascular mortality [39]. Other antihypertensives were added at the physician’s discretion if the four-drug combination was insufficient.

The number of patients adhering to the ABCD strategy was estimated from prescription records. The 5-year blood pressure control rates were calculated from a randomly selected subgroup of 500 outpatients.

### Ethical considerations

In this retrospective cohort study, data obtained from medical records were evaluated. Explanation on and consent regarding antihypertensive drug selection were verbally provided and sought, respectively, as part of routine care; hence, no interventions or formal consent were required for this study. The Tokyo Jikei University School of Medicine Ethics Committee waived the requirement for informed consent (approval no. 34–413 (11570)).

## Results

Approximately 2,000 patients with hypertension who visited the Tsunan Town Hospital between 1987 and 2016 (approximately 17% of the town’s population) were included in the study. Over the 30-year follow-up, the sample size varied by year, making precise tracking challenging. Overall, 2,028 patients were included when the study began; however, only 1,864 remained at the end of the study. All individual data were collected and analyzed by the Ministry of Health, Labor and Welfare. The researchers accessed the data for research purposes between November 15, 2015, and September 20, 2020. Data obtained between March 15, 2021, and May 22, 2021, were used in this study.

### Blood pressure target achievement rates

The achievement of blood pressure targets was assessed using a randomly selected subgroup of 500 outpatients. In period 0 (1987–1991), approximately 50% of the patients in the clinic achieved the blood pressure targets. In periods 1 and 2, using early-morning home blood pressure measurements, the achievement rates with two-drug combination therapy were 50% and 52%, respectively. The rates were 63% and 65%, respectively, when up to four-drug combinations were included. In periods 3, 4, and 5, the achievement rates with up to four drugs remained >60%: 63%, 67%, and 61%, respectively.

### Trends in antihypertensive drug usage

Nationwide, the combined use rate of ACE inhibitors and β-blockers decreased from 41.4% in period 0 to 38.5% in period 2 and further declined to 14.6% in period 5. In contrast, at Tsunan Town Hospital, the rate increased from 40.1% in period 0 to 64.6% in period 2 but subsequently declined to 47.6% in period 5. This trend was considered to indicate that after 2007, the choice of antihypertensive agents was entrusted to the succeeding physician.

Nationwide, ARB usage increased, whereas CCB usage decreased from 45.8% in period 0 to 20.1% in period 5. At the Tsunan Hospital, CCB usage was 51.6% in period 0, decreasing to 16.9% in period 2, and subsequently increasing to approximately 22.0% in period 5 (Table 1).

**Table 1:** Drug use proportions in Japan and Tsunan hospital across different years.

	Year	ACEI (%)	BB (%)	CCB (%)	ARB (%)	Others (%)
**Japan**	**1991**	23.9	17.5	45.8	0	12.7
	**1998**	24.8	13.7	50.7	0.7	10.1
	**2014**	1.3	7.3	20.1	64	7.3
**Tsunan**	**1991**	10.1	30	51.6	0	8.3
	**1998**	26.3	38.3	16.9	0.9	17.3
	**2014**	25.9	21.7	22	16.8	13.6

ACEI: angiotensin-converting enzyme inhibitors; BB: β-blockers; CCB: calcium channel blockers; ARB: angiotensin II receptor blockers; Others: other medications.

### Trends in SMRs in Tsunan

Table 2 presents the SMRs (with 95% CIs) for all causes and the three major diseases (heart disease, cerebrovascular disease, and malignant neoplasms) for period 0–5, spanning from 1987 to 2016.

**Table 2:** Trends in SMRs (95% confidence interval) for all deaths, malignant neoplasms, heart disease (ICD-10: I20–I52), and cerebrovascular disease.

	Period 0: 1987–1991	Period I: 1992–1996	Period II: 1997–2001	Period III: 2002–2006	Period IV: 2007–2011	Period V: 2012–2016
**All deaths**						
Japan	1.00	1.00	1.00	1.00	1.00	1.00
Tsunan Town						
- Total	0.94 (0.88–1.01)	0.88 (0.79–0.97)	0.80 (0.74–0.86)	0.87 (0.81–0.93)	0.78 (0.73–0.84)	0.84 (0.78–0.89)
- Male	0.93 (0.84–1.03)	0.91 (0.83–1.00)	0.82 (0.73–0.90)	0.95 (0.83–1.04)	0.86 (0.77–0.94)	0.88 (0.80–0.96)
- Female	0.95 (0.85–1.05)	0.88 (0.79–0.97)	0.78 (0.69–0.86)	0.78 (0.70–0.86)	0.72 (0.65–0.79)	0.80 (0.73–0.87)
**Malignant neoplasm**						
Japan	1.00	1.00	1.00	1.00	1.00	1.00
Tsunan Town						
- Total	0.86 (0.73–1.00)	0.78 (0.69–0.80)	0.68 (0.57–0.78)	0.87 (0.76–0.99)	0.84 (0.73–0.95)	0.95 (0.83–1.07)
- Male	0.86 (0.70–1.03)	0.94 (0.81–1.07)	0.69 (0.56–0.83)	0.96 (0.80–1.12)	0.92 (0.77–1.08)	0.92 (0.76–1.08)
- Female	0.86 (0.65–1.07)	0.83 (0.68–0.97)	0.64 (0.48–0.80)	0.75 (0.59–0.92)	0.73 (0.57–0.88)	0.99 (0.80–1.17)
**Heart disease**						
Japan	1.00	1.00	1.00	1.00	1.00	1.00
Tsunan Town						
- Total	0.91 (0.77–1.05)	0.77 (0.57–0.97)	0.55 (0.43–0.68)	0.73 (0.60–0.87)	0.72 (0.60–0.84)	0.81 (0.68–0.94)
- Male	0.87 (0.67–1.07)	0.88 (0.70–1.08)	0.52 (0.35–0.69)	0.76 (0.56–0.97)	0.78 (0.58–0.99)	0.87 (0.68–1.12)
- Female	0.94 (0.74–1.14)	0.82 (0.64–0.99)	0.58 (0.41–0.75)	0.71 (0.53–0.88)	0.68 (0.52–0.83)	0.75 (0.59–0.91)
**Cerebrovascular disease**						
Japan	1.00	1.00	1.00	1.00	1.00	1.00
Tsunan Town						
- Total	1.08 (0.89–1.26)	0.90 (0.66–1.13)	0.88 (0.72–1.04)	0.88 (0.72–1.05)	0.88 (0.71–1.05)	1.21(0.99–1.40)
- Male	1.08 (0.81–1.35)	0.97 (0.79–1.16)	1.00 (0.75–1.25)	1.16 (0.88–1.45)	0.89 (0.62–1.15)	1.14 (0.81–1.46)
- Female	1.07 (0.82–1.32)	1.13 (0.92–1.33)	0.77 (0.57–0.98)	0.65 (0.46–0.85)	0.88 (0.66–1.11)	1.25 (0.97–1.54)

SMR, standardized mortality ratio (with the nationwide average set at 1.00).

The changes in the all-cause SMR in Tsunan Town were as follows: period 0: 0.94 → period 1: 0.88 → period 2: 0.80 → period 3: 0.87 → period 4: 0.78 → period 5: 0.84.

Meanwhile, the shift in the SMR for deaths due to heart disease was as follows: Period 0: 0.91 → Period 1: 0.77 → Period 2: 0.55 → Period 3: 0.73 → Period 4: 0.72 → Period 5: 0.81.

The SMRs for all-cause and heart disease mortality began to show a significant decline below the national average from 1992 (Period 1), with the greatest decrease observed in Period 2. However, no significant fluctuations were observed in the SMR for cerebrovascular disease, and no significant differences from the national average were observed throughout the study period.

**Fig 1:**
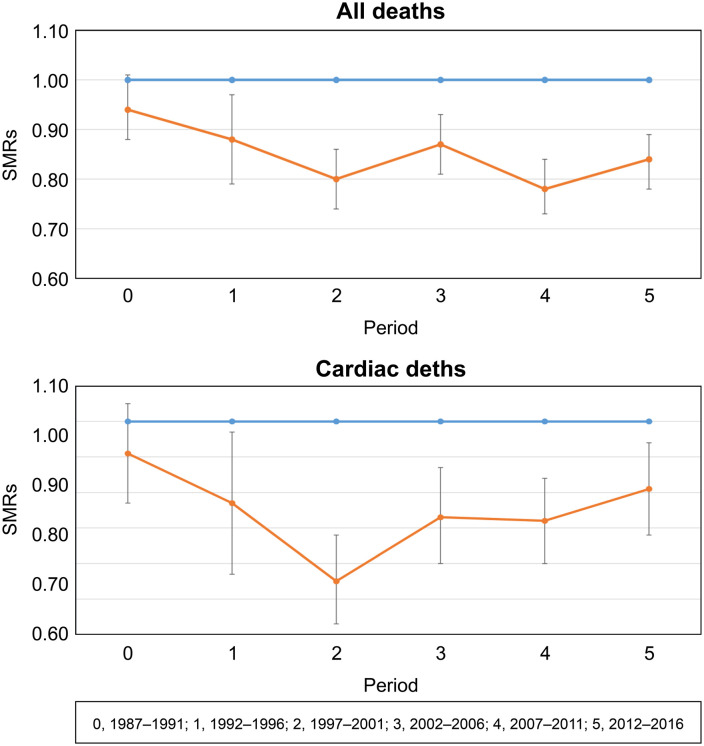
Five-year Trends in SMR for All-cause and Heart Disease Mortality (ICD-10: I20–I52). SMR, standardized mortality ratio. Blue line represents the entire country; Orange line represents Tsunan.

Fig 1 presents the trends in the 5-year SMRs for all-cause and heart disease mortality. The SMR for heart disease was lowest in period 2 (1997–2001), and although it has shown an upward trend since then, it remains at a low level.

## Discussion

In this study, we examined the impact of antihypertensive medications on all-cause mortality, particularly mortality due to heart disease, to indirectly test the hypothesis that these treatments influence the activation of neuroendocrine factors.

Compared with nationwide data from Japan, the all-cause mortality rate in Tsunan showed no significant difference from the national average in Period 0 (before 1991) but significantly declined throughout subsequent periods.

Similarly, the prevalence of cardiac mortality in Tsunan declined after the introduction of combined therapy with ACE inhibitors and β-blockers in 1992 (Period 1), and an association was observed between this combination therapy and reduced mortality rate. However, more rigorous studies are required to establish causality. The reduction in all-cause mortality rate observed in this study may be partly attributable to the decrease in cardiac mortality rate; however, since blood pressure control rates were comparable to the national average, it is conceivable that not only blood pressure reduction itself but also the pharmacological properties of the agents used may have influenced the outcomes.

Regarding the trend in reduced cardiac deaths, the greatest decline was observed in Period 2, when the combined use of ACE inhibitors and β-blockers increased. However, in Period 5, the decrease rate became smaller because of changes in antihypertensive drug usage ratios, such as reduced use of ACE inhibitors and β-blockers, alongside increased use of CCBs and ARBs. Nevertheless, the sustained effect of this therapy after 2007 may be considered a legacy effect resulting from delayed cardiovascular remodeling.

Reports from three recent intervention trials showed that adequate blood pressure reduction is a necessary and sufficient condition for cardiovascular event prevention [40–42]. However, the results on fatal endpoints (cardiovascular death and all-cause mortality) were not consistent. Specifically, significant reductions were found in SPRINT and ESPRIT, while STEP showed significance in only the primary composite endpoint that included alternative endpoints, such as revascularization, acute coronary syndrome, and hospitalization for heart failure, but not in fatal endpoints. Various classes of antihypertensive drugs were used in the former trials, including ACE inhibitors and β-blockers, whereas ARBs, CCBs, and diuretics were primarily used in the latter trial, indicating that while any antihypertensive medication may achieve the target blood pressure, the choice of drug is important to reduce fatal events. This observation supports the findings of the present study.

Our study is a cohort study based on real-world clinical data and likely included many high-risk patients undergoing secondary prevention of heart disease mortality and presenting with comorbidities, such as diabetes and dyslipidemia. Combining ACE inhibitors and β-blockers for these patients was particularly effective.

Conversely, the SMR for cerebrovascular disease in Tsunan showed no significant difference from the national average throughout the study period, suggesting that cerebrovascular mortality depends more on the degree of blood pressure reduction than on the type of antihypertensive drug used. This finding is consistent with reports from many previous intervention studies.

Intervention studies targeting patients with heart failure have shown that ACE inhibitors [43] and β-blockers [17] improve prognosis, and their combination is important [18]. However, intervention studies in which CCBs or diuretics were used have not shown improvements in prognosis [44,45]. These differences are believed to be due to differing effects on the activation of neuroendocrine factors [46,47].

The results of this study show that suppressing neuroendocrine activation is also important in antihypertensive treatment. Future prospective intervention studies comparing antihypertensive drug groups that suppress neuroendocrine factors with those that do not are warranted. Such research is particularly important for patients at high risk of death from heart disease.

The observed reduction in all-cause mortality may also be related to decreased mortality from malignant neoplasms. ACE inhibitors and β-blockers tend to reduce cancer-related mortality, whereas CCBs and diuretics have been reported to have the opposite effect [48]. Further investigation is needed to clarify the impact of antihypertensive therapy on malignancy and how cancer-related deaths have contributed to the results of this study.

### Limitations

This study mainly relied on clinical diagnostic data recorded on death certificates. The number of deaths was accurate; nonetheless, cause-of-death determination was not based on autopsy, and thus, objective reliability cannot be guaranteed. Additionally, the prevalence of comorbid conditions influencing mortality (e.g., heart failure, ischemic heart disease, diabetes, dyslipidemia, and smoking) should have been considered as confounders. However, longitudinal data on these factors during the follow-up were unavailable.

Given the retrospective observational nature of this study, we could not fully adjust for individual confounding factors, which represents an important limitation. Therefore, caution is warranted when interpreting the results. However, there were no substantial discrepancies in age and sex distributions (S1 Table), and the use of SMR allowed us to adjust for differences in age structure between groups, thus mitigating age-related bias in comparison with national data.

Finally, medical visits of a small number of patients with hypertension who received care outside Tsunan Town were not tracked.

### Conclusion

While this study was a retrospective observational study and thus has limitations in establishing causal relationships, it suggests that long-term (over 30 years) combination therapy with ACE inhibitors and β-blockers in the general population may have contributed to the reduction of all-cause and cardiovascular mortality rates among patients with hypertension. These findings provide important evidence for generating the hypothesis regarding the utility of antihypertensive therapy while considering neurohumoral factors. Based on these observational findings, it would be desirable to design and conduct interventional studies aimed at suppressing neurohumoral factors, and this study is considered to contribute to hypothesis generation for such future research.

## Supporting information

S1 Table Changes in the population structure of Tsunan town and Japan. (DOCX)
