# Correction: Antihypertensive therapy to prevent cardiac death: A study of combined ACE inhibitors and β-blockers—a retrospective cohort study in Tsunan Town, Japan

**DOI:** 10.1371/journal.pone.0340417

**Published:** 2026-01-05

**Authors:** Shinichiro Ishikawa, Yusaku Hayashi, Toshiyuki Abe, Susumu Tanaka

In Fig 1, the label “Cardiac Deths” should have been “Cardiac Death”. Please see the correct Fig 1 here.

**Fig 1:**
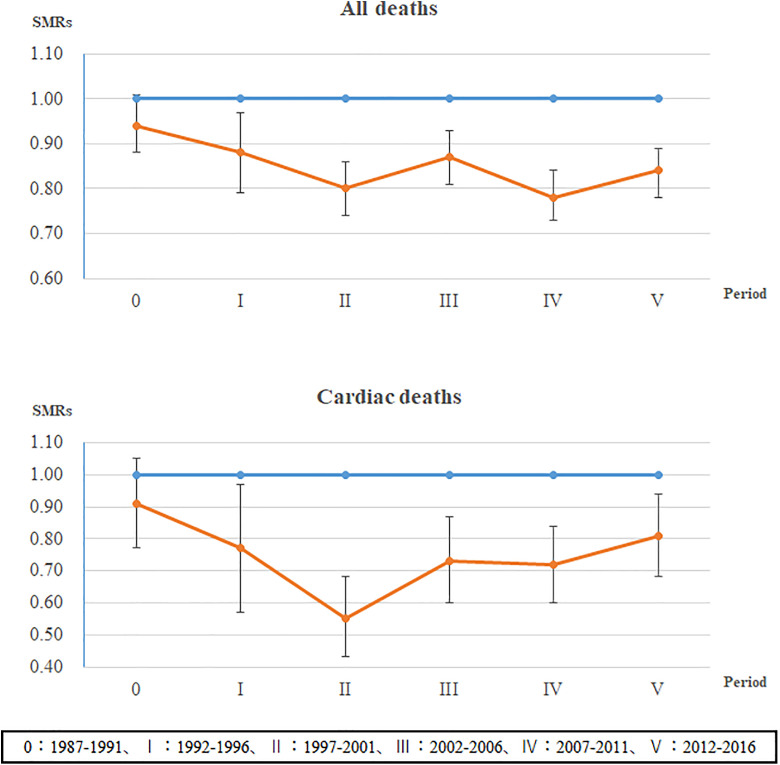
Five-year Trends in SMR for All-cause and Heart Disease Mortality (ICD-10: I20–I52). SMR, standardized mortality ratio. Blue line represents the entire country; Orange line represents Tsunan.

In the Acknowledgement statement, the name“T. Yasuzawa” should have been “T. Anzawa”. The correct statement is: We express our deepest gratitude to M. Tsuchiya, A. Sasaki, T. Sakamoto, S. Nakata, H. Suzuki, T. Hanafusa, K. Miyoshi, Y. Tanaka, F. Okazaki, T. Anzawa, A. Matsuyama, M. Uemura, T. Ito, and J. Koga for their cooperation in conducting this study. We would also like to thank Editage (www.editage.jp) for the English language editing.
