# Supplementary material for: Antihypertensive therapy to prevent cardiac death: A study of combined ACE inhibitors and β-blockers—a retrospective cohort study in Tsunan Town, Japan
Source: PLoS One. 2025 Oct 24;20(10):e0328142. doi: 10.1371/journal.pone.0328142 (PMC12551857; doi:10.1371/journal.pone.0328142)
Supplement: S1 Table — (DOCX) [file pone.0328142.s001.docx]

| **Japan(National)** | | | | | | | |
| --- | --- | --- | --- | --- | --- | --- | --- |
| Year | | 1990 | 1995 | 2000 | 2005 | 2010 | 2015 |
| Sex | Age group | Number(%) | Number(%) | Number(%) | Number(%) | Number(%) | Number(%) |
|  | Total | 123611167 | 125570246 | 126925843 | 127767994 | 128057352 | 127094745 |
|  | 15 years> | 22486239(18.2) | 20013730(16.0) | 18472499(14.6) | 17521234(13.8) | 16803444(13.2) | 15886810(12.6) |
|  | 15-64 years | 85903976(69.7) | 87164721(69.5) | 86219631(68.1) | 84092414(66.1) | 81031800(63.8) | 76288736(60.7) |
|  | 65 years< | 14894595(12.2) | 18260822(14.6) | 22005152(17.4) | 25672005(16.5) | 29245685(23.0) | 33465441(26.6) |
| Male | Total | 60696724(49.1) | 61574398(49.0) | 62110764(49.0) | 62348977(48.8) | 62327737(48.7) | 61841738(48.7) |
|  | 15 years> | 11517752(9.34) | 10246810(8.2) | 9459102(7.5) | 8971683(7.0) | 8602329(6.8) | 8133536(6.5) |
|  | 15-64 years | 42968512(34.9) | 43734829(34.9) | 43281355(34.2) | 42210963(33.2) | 40684202(32.0) | 38394322(30.6) |
|  | 65 years< | 5987637(4.86) | 7504253(6.0) | 9222116(7.3) | 10874599(8.5) | 12470412(9.8) | 14485469(11.5) |
| Female | Total | 62914443(50.9) | 63995848(51.0) | 64815079(51.0) | 65419017(51.2) | 65729615(51.3) | 65253007(51.3) |
|  | 15 years> | 10968487(8.9) | 9766920(7.8) | 9013397(7.1) | 8549551(6.7) | 8201115(6.5) | 7753274(6.2) |
|  | 15-64 years | 42935464(34.8) | 43429892(34.6) | 42938276(33.9) | 41881451(32.9) | 40347598(31.7) | 37894414(30.2) |
|  | 65 years< | 8906958(7.2) | 10756569(8.6) | 12783036(10.0) | 14797406(11.6) | 16775273(13.2) | 18979972(15.1) |
|  |  |  |  |  |  |  |  |
| **Tsunan Town** | | | | | | | |
| Year | | 1990 | 1995 | 2000 | 2005 | 2010 | 2015 |
| Sex | Age group | Number(%) | Number(%) | Number(%) | Number(%) | Number(%) | Number(%) |
|  | Total | 12955 | 12865 | 12389 | 11719 | 10881 | 10029 |
|  | 15 years> | 2281(17.6) | 2068(16.1) | 1811(14.6) | 1512(12.9) | 1185(10.9) | 989(9.9) |
|  | 15-64 years | 7694(59.4) | 7200(56.0) | 6594(53.2) | 6082(51.9) | 5624(51.7) | 5131(51.2) |
|  | 65 years< | 2980(23.0) | 3597(28.0) | 3984(32.2) | 4125(35.2) | 4062(37.4) | 3909(39.0) |
| Male | Total | 6346(49.0) | 6325(49.2) | 6047(48.8) | 5648(48.2) | 5221(48.0) | 4810(48.0) |
|  | 15 years> | 1176(18.5) | 1097(17.3) | 965(16.0) | 794(14.1) | 615(11.8) | 509(10.6) |
|  | 15-64 years | 3953(62.3) | 3731(59.0) | 3456(57.2) | 3203(56.7) | 2982(57.2) | 2703(56.2) |
|  | 65 years< | 1217(19.2) | 1497(23.7) | 1626(26.9) | 1651(29.2) | 1616(31.0) | 1598(33.2) |
| Female | Total | 6609(51.0) | 6540(50.8) | 6342(51.2) | 6071(51.8) | 5660(52.0) | 5219(52.0) |
|  | 15 years> | 1105(16.7) | 971(14.8) | 846(13.3) | 718(11.8) | 570(10.1) | 480(9.2) |
|  | 15-64 years | 3741(56.6) | 3469(53.0) | 3138(49.5) | 2879(47.4) | 2642(46.7) | 2428(46.5) |
|  | 65 years< | 1763(26.7) | 2100(32.1) | 2358(37.2) | 2474(40.8) | 2446(43.2) | 2311(44.3) |

**S1 Table. Changes in the population structure of Tsunan town and Japan**
